# Rapid identification of bacterial pathogens using a PCR- and microarray-based assay

**DOI:** 10.1186/1471-2180-9-161

**Published:** 2009-08-10

**Authors:** Anna-Kaarina Järvinen, Sanna Laakso, Pasi Piiparinen, Anne Aittakorpi, Merja Lindfors, Laura Huopaniemi, Heli Piiparinen, Minna Mäki

**Affiliations:** 1 Mobidiag Ltd, 00290 Helsinki, Finland

## Abstract

**Background:**

During the course of a bacterial infection, the rapid identification of the causative agent(s) is necessary for the determination of effective treatment options. We have developed a method based on a modified broad-range PCR and an oligonucleotide microarray for the simultaneous detection and identification of 12 bacterial pathogens at the species level. The broad-range PCR primer mixture was designed using conserved regions of the bacterial topoisomerase genes *gyrB *and *parE*. The primer design allowed the use of a novel DNA amplification method, which produced labeled, single-stranded DNA suitable for microarray hybridization. The probes on the microarray were designed from the alignments of species- or genus-specific variable regions of the *gyrB *and *parE *genes flanked by the primers. We included *mecA*-specific primers and probes in the same assay to indicate the presence of methicillin resistance in the bacterial species. The feasibility of this assay in routine diagnostic testing was evaluated using 146 blood culture positive and 40 blood culture negative samples.

**Results:**

Comparison of our results with those of a conventional culture-based method revealed a sensitivity of 96% (initial sensitivity of 82%) and specificity of 98%. Furthermore, only one cross-reaction was observed upon investigating 102 culture isolates from 70 untargeted bacteria. The total assay time was only three hours, including the time required for the DNA extraction, PCR and microarray steps in sequence.

**Conclusion:**

The assay rapidly provides reliable data, which can guide optimal antimicrobial treatment decisions in a timely manner.

## Background

Conventional diagnosis of a bacterial infection mainly relies on culture-based testing. These cultivations usually yield diagnostic results in days or in some cases up to a week after sampling. Furthermore, cultivation of bacteria is not always successful under laboratory conditions. Such failures may occur due to unsuitable culturing conditions and methods for the bacterial species in question. Alternatively, the particular patient under investigation may have received antimicrobial therapy before sampling. Molecular methods based on nucleic acid amplification and hybridization aim to circumvent these problems and hasten diagnostic procedures. In such methods, the pathogen is simultaneously detected and identified, which results in more rapid diagnoses than those obtained by conventional culturing methods and obviates the need for additional culture tests. Rapid diagnostics can also reduce the use of antimicrobial agents in addition to allowing a faster switch to the most optimum treatment, thus reducing both side-effects and costs [1,2].

Microarrays allow the hybridization-based detection of multiple targets in a single experiment. Arrays have mostly been utilized in gene expression profiling. However, the use of microarrays in microbial diagnostics has been recently reviewed by Bodrossy and Sessitsch (2004) [3]. Roth and co-workers (2004) [4] described the diagnostic oligonucleotide array targeting species-specific variable regions of the topoisomerases genes *gyrB *and *parE *of respiratory bacterial pathogens. These authors used a broad-range polymerase chain reaction (PCR) method, which is based on the primers that recognize conserved sequences of genes that encode essential molecules. The most common bacterial broad-range PCR methods use primers that recognize conserved DNA sequences of bacterial genes that encode ribosomal RNA (rRNA 16S or 23S). However, resolution problems at the genus and/or species level occur when distinguishing between closely related bacterial species solely by their conserved 16S rDNA sequences. Moreover, the sequencing of the whole 16S rRNA gene is recommended for reliable microbial speciation [5]. In comparison, the *gyrB *gene discriminates between related bacterial species more precisely than the 16S rRNA gene, which makes it a more suitable gene for such species identification [6,7].

In addition to identifying the causative pathogen of the infection, the rapid identification of antimicrobial resistance markers can further guide and, if necessary, re-direct the appropriate treatment. Methicillin resistant *Staphylococcus aureus *(MRSA) is one of the common pathogens responsible for nosocomial infections. Furthermore, among coagulase-negative staphylococci (CNS), methicillin resistance is prevalent [8]. Methicillin resistance in *Staphylococcus *species arises principally by the acquisition of a large genetic element, the staphylococcal cassette chromosome, SCC*mec *[9]. The SCC*mec *carries the *mecA *gene, which encodes penicillin binding protein PBP2a, the main causal factor of methicillin resistance. Different types of SCC*mec *cassettes and their variants have been identified [10,11]. The current methods for MRSA detection are based on either the phenotypic expression such as oxacillin resistance, or genotypic characterization.

For this study, we used modified broad-range PCR primers that originate from the conserved regions of genes that encode the topoisomerases together with specific oligonucleotide probes located at hyper-variable regions flanked by the primers. Using these primers and probes, single or even multiple infection-causing bacteria could be simultaneously detected and identified. The bacterial pathogen panel of the assay covered the following species: *Acinetobacter baumannii*, *Enterococcus faecalis, Enterococcus faecium*, *Haemophilus influenzae, Klebsiella pneumoniae*, *Listeria monocytogenes, Neisseria meningitidis, Staphylococcus aureus, Staphylococcus epidermidis*, *Streptococcus agalactiae, Streptococcus pneumoniae*, *Streptococcus pyogenes *and selected CNS species. These bacteria are examples of highly virulent, potentially multi-antimicrobial resistant or the most common etiologic agents associated with various life-threatening conditions. Such conditions include: sepsis, infective endocarditis and central nervous system infection. All these conditions necessitate rapid and accurate diagnostics to improve the chances of a positive outcome for the patient. We used the ArrayTube™ as a microarray platform for the probes. The ArrayTube™ has been demonstrated to detect and identify bacterial pathogens with a high degree of sensitivity [12-14], differentiate between various pathotypes of the same bacterial species [15] and to be capable of detecting antimicrobial resistance genes [16] from an isolated DNA sample. Furthermore, by including specific primers and probes for the *mecA *methicillin resistance gene in the same assay, we were able to associate the *mecA *gene with a particular *Staphylococcus *species present in the sample. The combination of broad-range PCR and array-based methods provided a sensitive and specific approach for detecting and identifying bacterial pathogens along with finding possible resistance markers.

## Results

### Assay design

**Figure 1:**
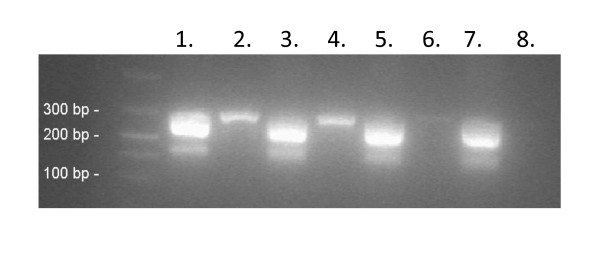
**Comparison of the amplification efficacy between the *gyrB/parE *primer pairs of this study (lanes 1, 3, 5, and 7) and those of Roth *et al*., (2004) **[4]** (lanes 2, 4, 6, and 8)**. The production of ssDNA during the PCR program are shown with the species of *E. faecalis *(lane 1 and 2), *E. faecium *(lane 3 and 4). *K. pneumoniae *(lane 5 and 6), and *N. meningitidis *(lane 7 and 8) by gel electrophoresis using a 2% agarose gel containing SYBR^® ^Green II. The ssDNA amplicons of *gyrB/parE *(200 bp) were detected using the primer pair of this study together with the dsDNA amplicons of *gyrB/parE *(300 bp).

First, we re-designed and modified the bacterial broad-range *gyrB/parE *primers [4] by using inosines to reduce the level of degeneration. These modifications also facilitated the use of a novel PCR method for the assay (PCR program described in Materials and Methods). The PCR method had two distinct phases: a three-step PCR phase that exponentially produced dsDNA, followed by a two-step PCR phase that took place under two different conditions and which produced ssDNA in a linear manner. The method is based on partly overlapping annealing temperatures of the forward and reverse primers. After the PCR step, amplicons were directly, without any denaturation step, used in hybridization, due to the production of ssDNA during the second PCR phase. This avoided the problems resulting from suboptimal or unreliable denaturation associated with standard PCR methods. The effectiveness of the re-designed *gyrB/parE *primers and the production of ssDNA during the PCR step were assessed using DNA extracts of various bacterial species. Figure 1 shows the production of ssDNA and the same or even improved sensitivity for bacteria included in the assay panel.

When designing the microarray probes for *A. baumannii*, *E. faecalis, E. faecium*, *H. influenzae, K. pneumoniae*, *L. monocytogenes, N. meningitidis, S. aureus, S. epidermidis*, *S. agalactiae, S. pneumoniae*, *S. pyogenes*, and the selected CNS species, we used the *gyrB *and *parE *sequences of these bacteria together with those of other clinically relevant bacteria. The sequence alignments were used to maximize the specific hybridization of the consensus sequences of the targeted bacteria, while minimizing the cross-hybridization of sequences of any non-targeted bacteria. Various *in silico *parameters were used in the design process to assess the accuracy of the oligonucleotide probes. Annealing potential was predicted by calculating the thermodynamic factors, whereas sequence specificity was evaluated by sequence comparisons and homologue searches of the EBI and NCBI databases using the BLAST algorithm. The oligonucleotide probes for the final microarray layout (Table 1) were chosen from a set of oligonucleotide probes tested in the laboratory.

**Table 1:** Oligonucleotide probes included in the final microarray layout.

Targeted bacteria	Sequence (5'->3')	Length	**T_m _**(°C)
*Acinetobacter baumannii*	AGTGTTTCAGATAATGGCC	19	47

	AATTATTGTCACGATTCACGAGG	23	52

			

*Enterococcus faecalis*	CTGGTATCCCAACAGTAGAAGTA (*parE*)	23	53

			

*Enterococcus faecium*	TATCACCGTTATTGACGACGGTC	23	55

	GGATACCTGTAGATATCCAGGCA	23	55

			

*Haemophilus influenzae*	GGCCATTGTTCCGATATTATCG	22	53

	GTTCCGATATTATCGTGACAA	21	49

			

*Klebsiella pneumoniae*	TACTGCAAAGATATCGTTGTCA	22	49

			

*Listeria monocytogenes*	TACAATCGAAGCTGATAACAGCA	23	52

	ACTGTTCGTGATAACGGACGTGG	23	57

	GGTCGTCCAACAGTAGAAGT	20	52

			

*Neisseria meningitidis*	AATCACGGTAACGATACACGC	21	52

			

*Staphylococcus aureus*	CTGTCGAAGTTATTTTAACTGTTT	24	49

	AATAGTATCGATGAAGCATTAGCTG	25	53

			

*Staphylococcus epidermidis*	TAGTCATATTGAAGTTRTAATTGAG	25	48–49

	CATTAGCAGGTTATGCTAGTCATA	24	52

			

CNS	TCAACTTCAGAAAAAGGTTTACA	23	48

	CGCCCAGCAGTTGAAGTTATCT	22	55

			

*Streptococcus agalactiae*	TTACATTGAACCAGATAACTCTA	23	48

	TGGAAGACCAGCTGTAGAGACAG	23	57

			

*Streptococcus pneumoniae*	TGGTGATCGTATTGATGTAACTA (*parE*)	23	50

			

*Streptococcus pyogenes*	GTCCCGCCGTTGAAACAGTT	20	54

	TTTTTACAGTCTTACACGCAGGT	23	52

	GCAGGTTTTGCCTCTCATATTAAAGTCTT	29	57

			

*mecA*	TGATTATGGCTCAGGTACTGC	21	52

	TGGCTCAGGTACTGCTATCCA	21	54

	ATTAGCACTTGTAAGCACACC	21	50

	TACTGCTATCCACCCTCAAAC	21	52

If not otherwise stated, the presented sequence targets the *gyrB *gene.

Melting temperature (T_m_, basic) is calculated using software available at http://www.basic.northwestern.edu/biotools/oligocalc.html.

**Figure 2:**
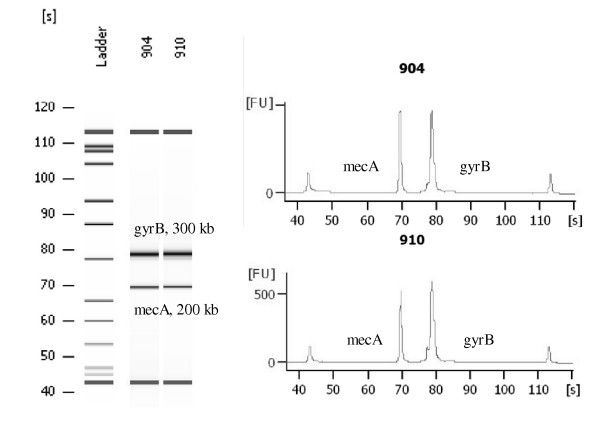
**Multiplex amplification of *gyrB *and *mecA *visualized by electropherograms (Agilent Technologies 2100 Bioanalyzer) in two MRSA clinical isolates**. X-axis presents time (s) and Y-axis presents the amount of fluorescence (FU).

We added an option for molecular identification of methicillin resistant *Staphylococcus *species by including the methicillin resistance gene *mecA *in the assay. The identification was based on multiplex PCR amplification of the *gyrB/parE *and *mecA *gene fragments (Figure 2). We then detected the presence of amplified *S. aureus *or *S. epidermidis *DNA on the microarray by using species-specific probes. The presence of coagulase negative staphylococcal DNA other than that associated with *S. epidermidis *was detected by genus-specific probes. The presence of the ~200 bp *mecA *PCR product was indicated by the *mecA *probes. Thus, when the *mecA *association was correlated with *Staphylococcus aureus*, *Staphylococcus epidermidis*, and CNS detection, information about the methicillin resistance of staphylococci was provided.

### Analysis of Staphylococcus species on the array

Because the only probes covering multiple bacterial species in the assay were the CNS probes, we investigated in detail the coverage and specificity of our *Staphylococcus *panel including probes for *Staphylococcus aureus*, *Staphylococcus epidermidis*, and CNS species (Table 1). The CNS-specific probes systematically detected specific staphylococcal species including *S. xylosus*, *S. haemolyticus*, *S. saprophyticus*, and *S. lugdunensis*. However, some other clinically relevant *Staphylococcal *species, such as *S. capitis*, *S. cohnii*, *S. hominis*, *S. schleiferi*, and *S. warnerii *were not covered by the panel (Table 2).

**Table 2:** The species coverage of *Staphylococcus *probe panel.

Phenotypic identification	Number of strains	Positive identification on microarray	Negative identification on microarray
*S. capitis*	1		1

*S. cohnii*	1		1

*S. haemolyticus*	1	1	

*S. hominis*	2		2

*S. ludgunensis*	2	2	

*S. saprophyticus*	2	2	

*S. schleiferi*	1		1

*S. warnerii*	2		2

*S. xylosus*	2	2	

			

**TOTAL**	**14**	**7**	**7**


*S. epidermidis*	2	2	

*S. epidermidis + mecA*	2	2	


**TOTAL**	**4**	**4**	**0**

*S. aureus*	5	4	1 (2/4 probes identified)

*S. aureus + mecA*	3	3	

*S. intermedius*	1		1

			

**TOTAL**	**9**	**7**	**2**

**Figure 3:**
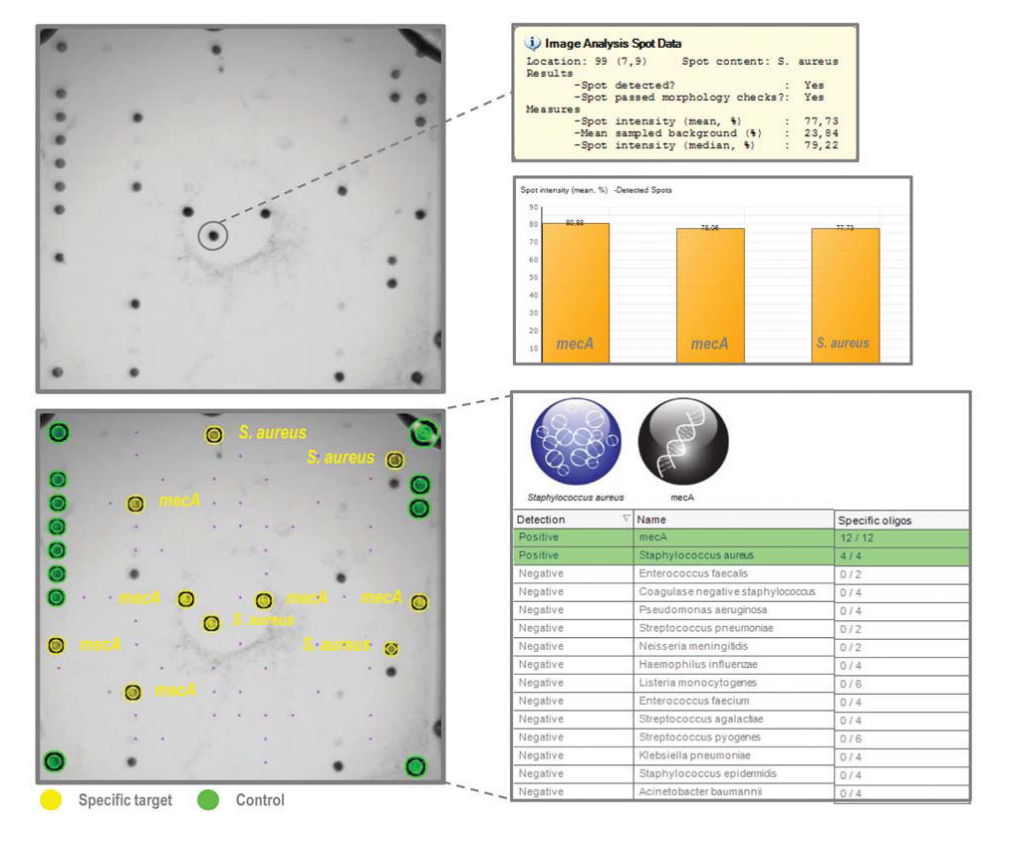
**Detection of methicillin resistant *Staphylococcus aureus *(MRSA) using the Prove-it™ Advisor software**. The original array image illustrates the positive hybridization of *Staphylococcus aureus *and *mecA *targets. The accompanied statistics are also visualized. In the processed image, yellow spots denote the identified target oligonucleotides and green spots the identified position control oligonucleotides. The unmarked visible spots are not included in the final array layout.

*S. epidermidis *had specific probes for identification, which functioned optimally. Similarly, the specific probes for *S. aureus *functioned well, with the exception of one *S. aureus *sample, which was not detected because only one of a duplicate set of oligonucleotide probes was identified. In the dataset, the *mecA *detection was associated with *S. epidermidis *and *S. aureus*. Figure 3 shows the representative hybridization result of MRSA clinical isolates, and illustrates the simultaneous detection of the *gyrB *and *mecA *targets. The hybridization results are displayed by the Prove-it™ Advisor software, which provides the original and analyzed array images, analyzed data and the accompanied statistics. The presence of *S. epidermidis *in a sample was reported by the Prove-it™ Advisor software when *S. epidermidis *specific probes were positive. According to the built-in identification rules of the software, a CNS positive finding would be reported when *S. epidermidis *specific probes remained negative.

### Evaluation of the specificity of the probes

To determine the wet-lab specificity of the oligonucleotide probes and any possible cross-hybridization that might lead to false positive bacterial identification, the sample material containing 102 clinical isolates of 70 untargeted bacteria (Table 3) were subjected to multiplex *gyrB/parE/mecA *PCR and subsequent hybridization on the microarray. In addition, specificity of dsDNA and ssDNA amplification was verified by gel electrophoresis. The bacterial panel under test covered a large number of clinically relevant bacterial species related to the targeted bacteria, such as *Streptococcus mitis*, a close relative of pneumococcus, and *Klebsiella oxytoca *and *Klebsiella pneumoniae *subsp. *ozeanae*, close relatives of *K. pneumoniae*, and also bacteria of normal flora, such as *Corynebacterium *and *Stomatococcus *species. No significant cross-hybridization occurred between any targets. Only one cross-hybridization led to a false positive identification: *Klebsiella pneumoniae *subsp. *ozeanae *was reported as *Klebsiella pneumoniae *subsp. *pneumoniae*.

**Table 3:** Results of specificity testing using clinical isolates and reference strains of untargeted bacteria.

Phenotypic identification	No of strains	Negative identification on microarray	Positive identification on microarray
*Actinobacillus actinomycetemcomitans*	2	2	

*Actinomyces *sp	1	1	

*Aerococcus viridans*	1	1	

*Aeromonas hydrophila*	1	1	

*Aeromonas sobria*	2	2	

*Alcaligenes xylosox*	1	1	

*Arcanobacterium haemolyticum*	2	2	

*Bacillus cereus*	2	2	

*Bacillus *sp	2	2	

*Bacteroides fragilis *group	2	2	

*Bacteroides ureolyticus*	1	1	

*Campylobacter fetus*	2	2	

*Campylobacter coli*	1	1	

*Campylobacter jejuni*	2	2	

*Capnocytophaga canimorsus*	1	1	

*Citrobacter diversus*	1	1	

*Clostridium clostridiumforme*	1	1	

*Clostridium perfringens*	1	1	

*Clostrium *sp	2	2	

*Corynebacterium jeikeium*	2	2	

*Corynebacterium *sp	2	2	

*Desulfovibrio *sp	2	2	

*Diphtheroid*	2	2	

*Enterobacter aerogenes*	1	1	

*Enterobacter cloacae*	2	2	

*Enterobacter hormaechei*	1	1	

*Enterobacter *sp	1	1	

*Enterococcus avium*	2	2	

*Enterococcus casseliflavus*	1	1	

*Eubacterium *sp	1	1	

*Fusobacterium nucleatum*	1	1	

*Hafnia alvei*	2	2	

*Klebsiella oxytoca*	2	2	

*Klebsiella ozeanae*	1		1

*Lactobacillus *sp	1	1	

*Lactococcus *sp	1	1	

*Leuconostoc *sp	2	2	

*Micromonas micros*	1	1	

*Moraxella catarrhalis*	2	2	

*Moraxella *sp	2	2	

*Morganella morganii*	2	2	

*Pantoea agglomerans*	1	1	

*Pantoea *sp	1	1	

*Peptostreptococcus *sp	1	1	

*Porphyromonas gingivalis*	1	1	

*Prevotella *sp	1	1	

*Propionibacterium acnes*	2	2	

*Proteus vulgaris*	1	1	

*Pseudomonas fluorecens*	1	1	

*Pseudomonas fluorecens/putida*	1	1	

*Pseudomonas oryzihabitans*	1	1	

*Pseudomonas stutzeri*	2	2	

*Rothia dentacariosa*	1	1	

*Salmonella enterica *subsp. *enterica*	2	2	

*Serratia liquifaciens*	1	1	

*Serratia marcescens*	2	2	

*Sphingomonas paucimobilis*	1	1	

*Stenotrophomonas maltophilia*	2	2	

*Stomatococcus mucilaginosus*	1	1	

*Stomatococcus *sp	1	1	

*Streptococcus anginosus*	2	2	

*Streptococcus bovis*	2	2	

*Streptococcus dysgalactiae *subsp. *equisimilis*	1	1	

*Streptococcus equisimilis*	1	1	

*Streptococcus *group G	2	2	

*Streptococcus mitis*	2	2	

*Streptococcus sanguis*	2	2	

*Streptococcus *sp	1	1	

*Streptococcus viridans*	2	2	

*Yersinia pseudotuberculosis*	1	1	

			

TOTAL	102	101	1

### Evaluation of the assay using the blood culture dataset

We received a randomly obtained set of 186 blood culture samples, including 146 blood culture positive and 40 blood culture negative samples, from the Department of Bacteriology, HUSLAB (Finland) to compare the clinical applicability of the assay with the standard methodology. Samples were analyzed using the following workflow: a) DNA was extracted using the easyMAG extraction device, b) multiplex PCR amplification was performed in a standard thermal cycler and the success of amplification was verified by gel electrophoresis, c) subsequent hybridization was performed on the microarray, d) and finally the analyses of the hybridization images and result reporting were conducted using the Prove-it™ Advisor software. The assay time for one sample or 24 samples, excluding time required for sample preparation prior to DNA extraction, was three or under four hours, respectively. The obtained results were compared to the blood culture results assessed by HUSLAB.

The DNA extraction and PCR controls included in each test series were required to be negative for the acceptance of a particular test series. Negative controls gave negative hybridization results. However, two of these samples could not be analyzed by software due to automatic gridding problems. As a consequence, they were analyzed manually. The target identification was interpreted using the specific built-in rules and parameters of the Prove-it™ Advisor software. Briefly, all oligonucleotide probes for the specific target including their duplicates were required to be positive, with the exception of the CNS probes of which two out of four probes were required for reporting a positive finding. Furthermore, if the threshold limits were not exceeded for the oligonucleotide probes being measured, the obtained negative result was considered as a true negative.

The identified bacteria are presented in Table 4. A total of 69 positive and 117 negative identifications were obtained. Nine targets from the pathogen panel were detected in the samples of which *S. aureus, E. faecalis*, and *S. epidermidis *occurred with the highest incidences. The other identified bacteria were *K. pneumoniae, S. pneumoniae, S. pyogenes, E. faecium, S. agalactiae *and CNS. Bacterial species included in the pathogen panel, but not present in the samples were *A. baumannii*, *H. influenzae, L. monocytogenes*, and *N. meningitidis*. A total of 32 different microbes were present in the blood culture positive samples, and none of these microbes caused false positive identifications through cross-hybridization. The correct negative result was achieved for numerous different pathogens including *Bacillus *sp., *Escherichia coli, Enterobacter cloacae*, *Salmonella enterica *subsp. *enterica, Streptococcus sanguis*, *Streptococcus bovis*, and *Candida albicans *(Table 4). All of the 40 blood culture negative samples analyzed by our assay were reported as negative.

**Table 4:** Pathogens identified from the blood culture samples using PCR- and microarray-based analysis.

Correct positive identification of the bacteria	Number	Correct negative identification	Number
*Staphylococcus aureus*	24	*Bacillus *sp	2

*Enterococcus faecalis*	9	*Bacteroides fragilis *group	2

*Staphylococcus epidermidis +mecA*	8	*Candida albicans*	4

*Klebsiella pneumoniae*	7	*Diphtheroid*	1

*Streptococcus pneumoniae*	6	*Enterobacter cloacae*	1

*Streptococcus pyogenes*	6	*Enterococcus casseliflavus*	1

*Enterococcus faecium*	4	*Enterococcus *sp	4

CNS (*Staphylococcus haemolyticus*)	1	*Escherichia coli*	19

CNS + *mecA *(*S. haemolyticus*)	1	*Escherichia coli, Streptococcus viridans*	2

*Streptococcus agalactiae*	1	*Fusobacterium necrophorum*	3

		*Fusobacterium nucleatum, Micromonas micros*	1

**Correct positive identification of the bacteria but an additional *mecA *marker identified**		*Klebsiella oxytoca*	4

*Streptococcus pneumoniae + mecA*	1	*Micrococcus *sp	1

*Enterococcus faecalis + mecA*	1	*Propionibacter *sp	2

		*Pseudomonas aeruginosa*	3

		*Pseudomonas*-like gram- rod	1

		*Salmonella *Enteritidis	3

		*Salmonella *Paratyphi A	1

		*Stenotrophomonas maltophilia*	1

		*Streptococcus betahemolytic *group C	1

		*Streptococcus bovis*	1

		*Streptococcus sanguis* (co-infection with *K. pneumoniae*)	

		*Streptococcus viridans*	4

		Blood culture negative samples	40

			

		False negative identification*	

		CNS	6 (0)

		*Staphylococcus aureus*	2 (1)

		*Staphylococcus epidermidis*	2 (1)

		*Streptococcus pyogenes*	1 (1)

		*Streptococcus agalactiae*	1 (0)

		*Streptococcus pneumoniae*	1 (0)

		*Enterococcus faecalis*	1 (0)

		*Enterococcus faecium*	1 (0)

* The figures obtained after post hoc adjustments are presented in parentheses

When comparing our data with those of the blood culture results, 17 discrepant results were observed, of which 15 were false negatives and two false positives. Six of the samples reported as false negatives contained *S. agalactiae*, *S. epidermidis*, *S. pneumoniae, E. faecalis*, *E. faecium*, and *S. aureus *as a causative agent. In these cases, the strict detection rules caused the final outcome to be below the level required for positive identification. These six false negatives were caused by either one completely missing or one low quality duplicated probe, giving results that were insufficient to meet the strict positive identification criteria. Therefore these samples were reported as negative findings by the Prove-it™ Advisor, although other duplicates and probes were detected. We noticed that by using less strict identification rules, these samples were identified correctly. Thus, these samples were considered to be true positives when calculating the final specificity and sensitivity values of the assay.

The other nine samples reported negative by the the Prove-it™ Advisor were: *S. pyogenes*, *S. aureus*, *S. epidermidis*, and six CNS samples. We sequenced the CNS samples using the 16S rRNA gene. Sequencing revealed that these unidentified CNS samples contained *S. pasteuri, S. capitis *and *S. hominis *(four samples). The *mecA *gene was identified in two of the CNS samples. The two positive *mecA *findings were associated with *S. capitis *and *S. hominis*. None of the species in the six CNS samples was covered by the CNS probes of the assay panel (Table 2), thus these samples were considered to be true negatives. The reasons for the remaining three false negative samples (*S. pyogenes*, *S. aureus*, *S. epidermidis*) remained undetermined. The samples were not amplified by the 16s rRNA PCR, suggesting that they could have contained PCR inhibitors or degraded DNA.

Two false positive results were observed due to the detection of the *mecA *gene marker associated with the non-staphylococcus causative agent *S. pneumoniae *and *E faecalis*. The causative agent was in line with the corresponding blood culture result.

When the results of the assay were compared with the identification provided by HUSLAB, a sensitivity of 82 percent and specificity of 98 percent were achieved. After the alterations presented above were implemented, the sensitivity increased to 96 percent while the specificity remained at 98 percent (Table 5).

**Table 5:** Comparison of the blood culture results with the PCR- and microarray-based analysis.

	Positive identification on microarray	Negative identification on microarray	
Positive identification by conventional methods	73	3	Sensitivity 96%^#^ (89.0–98.6)*

Negative/positive identification by conventional methods	2	108	Specificity 98% (93.6–99.5)*

* Calculations are conducted according to CLSI recommendations.

^# ^Initial sensitivity of 82% was observed.

## Discussion

Microarrays are widely used in gene expression and genotyping applications in research settings but their use in diagnostics is still rare. Nevertheless, microarray technology and DNA-based approaches are believed to have great clinical potential in the field of infectious diseases [17]. In this study, we described a combined PCR- and microarray-based assay for the rapid and reliable detection of *A. baumannii*, *E. faecalis, E. faecium*, *H. influenzae, K. pneumoniae*, *L. monocytogenes, N. meningitidis, S. aureus, S. epidermidis*, *S. agalactiae, S. pneumoniae*, *S. pyogenes *and selected CNS (non-*S. epidermidis*) species.

In this study, we introduced a novel multiplex-PCR method that first produces dsDNA exponentially, after which ssDNA is produced in a linear manner. During the linear phase, the high annealing temperature allows only the reverse primer to function due to the T_m _difference between forward and reverse primers. Thus the whole PCR procedure can be conveniently performed in a single multiplex PCR amplification reaction without manual involvement. In our method, sufficient quantities of ssDNA are produced during the PCR reaction. Consequently, the conventional methods such as alkali or heat treatment, or asymmetric PCR are rendered unnecessary for generating a single stranded target for microarray hybridization [18,19]. Our method, therefore, enables a rapid protocol for assay as hybridization can be performed immediately after the PCR step. A similar type of PCR method has been developed by Zhu *et al*. (2007) [20]. These authors used forward primers tagged with an unrelated universal sequence at the 5' end to create the necessary T_m _difference between the forward and reverse primer. In contrast to the method of Zhu *et al*. (2007) [20] the temperature difference in our method is achieved by target-specific primers that enable rapid PCR cycling. In this study, we used our method for the multiplex amplification of the *gyrB *and *mecA *genes.

The *gyrB *gene region has been shown to be capable of discrimination when identifying closely related bacterial species [6,7]. When the specificity of our assay was evaluated using nucleic acid from 70 different untargeted bacteria, only one cross-reaction was observed: *Klebsiella pneumoniae *subsp. *ozeanae *was reported as *Klebsiella pneumoniae *subsp. *pneumoniae*. In addition to the *gyrB *gene, the 16S rRNA gene has been used in bacterial speciation, partly due to the large number of microbial 16S rDNA sequences available in the public databases [5,21]. In this study, the 16S rRNA gene and the corresponding public databases were used to study objectively any discrepancies in bacterial identification between the compared methods.

Despite the selected gene, designing discriminating probes between closely related species can be demanding. Setting the appropriate threshold values and making identification rules for target detection are specific challenges, which can be overcome by the means of bioinformatics. In our study, the final identification of a bacterial pathogen was based on one to three different oligonucleotides on the microarray. All these were spotted as duplicates and all of which, with the exception of CNS, were required to pass threshold values set for their positive identification. When more pathogens are included on the array, the designing of the probes, the setting of threshold values [22], and formulation of identification rules will require intensive testing. The testing procedure can be enhanced by automated data analysis, which provides objective and reproducible interpretation of the results. In our study, the Prove-it™ Advisor software generated data analysis for reporting and allowed effective data management and tracking.

We evaluated the assay by comparing its results with those of sepsis diagnostics, although other applications using specimens from normally sterile site of the body are feasible as well. Our sample material consisted of 186 blood culture samples and causative agents were identified originally in 69 of these samples. These positives corresponded to nine of the targets on the assay pathogen panel. However, some of the targets in the pathogen panel, *A. baumannii*, *H. influenzae, L. monocytogenes*, and *N. meningitidis*, were not present in any of the samples and no false identifications of these bacteria were made. When comparing these data with those of the blood culture results, discrepancies were observed due to the limited numbers of CNS probes on the panel, or for unknown reasons. The CNS probes on the panel were selected to cover the two most clinically prevalent CNS species *S. haemolyticus *and *S. saprophyticus*, and the most virulent species *S. lugdunensis*. If more CNS species were needed to be covered by the assay, their respective probes could be designed and added to the CNS probe panel [23]. Such species could be *S. pasteuri, S. capitis *and *S. hominis *all three of which were present in the blood cultures analyzed in our study.

We encountered some challenges with reconciling the microarray image analyses data and building optimal detection rules for the precise identification of all the pathogens. These specific problems are illustrated by missing or suboptimal duplicates causing false negative identifications. The microarray image and data analysis present commonly acknowledged challenges, especially when the microarray data quality is not optimal. For instance, the distinction between the actual spots and artifacts on the array, or the gridding of the image can be problematic [24]. These challenges in automated image and data analyses together with result reporting could be a reason for the current lack of available microarray-based diagnostics. In this study, a few microarray images and results were interpreted manually to overcome the technical problems encountered. Of course, this would not be appropriate for a diagnostic assay, for which such post hoc adjustments could not be made.

In general, the adjusted results were in line with the conventional blood culturing method, regarded as a gold standard in sepsis diagnostics. Our data had a specificity of 98 percent and sensitivity of 96 percent (initial sensitivity of 82 percent). Similar results namely: a specificity of 100 percent for the genus level and 97 percent for the species level using reference strains and clinical isolates were reported by a comparable method [21].

Simultaneous early detection of antimicrobial resistance markers and the causative pathogen of an infection in a clinical setting can direct the antimicrobial treatment optimally [2]. In our study, we included the methicillin resistance gene *mecA *in the assay. As a consequence, the *mecA *findings were associated with the positive findings of *S. epidermidis *or other CNS bacteria. Two samples had non-staphylococci bacteria and these *mecA *findings were later indicated as positive for CNS (data not shown). In Finland, the prevalence of MRSA in bloodstream infections is low [25]. Therefore, no MRSA samples were included in the clinical samples. For this reason, our data demonstrate the combined detection of *S. aureus *and the *mecA *gene fragment with the clinical isolate of MRSA (Figure 3).

## Conclusion

Genotypic characterization of bacteria is advantageous when compared to phenotypic methods. The latter require a prolonged cultivation period for the suspected bacteria and pure bacterial cultures for various biochemical assays. The accurate detection of multiple pathogens and resistance markers simultaneously reduces the time needed to start effective antimicrobial treatment. We conclude that broad-range PCR amplification with subsequent hybridization on a microarray is a rapid diagnostic tool in identifying causative agents of bacterial infections in various specimens from normally sterile site of the body or non-cultured samples. In this study, we presented proof-of-concept for one combination of bacterial probes but depending on the clinical application, the assay could be modified to cover different species profiles.

## Methods

### Samples

#### Clinical isolates and reference strains for cross-hybridization studies

A total of 102 clinical isolates and reference strains of various bacteria from American Type Culture Collection (ATCC, VA), Deutsche Sammlung von Mikroorganismen und Zellkulturen (DSMZ, Germany), or Helsinki University Central Hospital Laboratory (HUSLAB, Finland) were used for the cross-hybridization comparisons. Bacteria were grown in cystine lactose-electrolyte-deficient (CLED), blood, or chocolate agar plates. Culturing was performed under aerobic or anaerobic conditions depending on the bacterial species. All strains were incubated at 37°C for at least for 24 hours.

#### Clinical isolates and reference strains of *Staphyloccus *species

The *Staphylococcus *samples comprised ATCC strains and clinical isolates that were characterized by conventional methods. A total of 18 CNS samples including *S. capitis *(ATCC27840), *S. cohnii *(ATCC29972), *S. haemolyticus *(one clinical isolate), *S. hominis *(ATCC25615, ATCC27844), *S. lugdunensis *(two clinical isolates), *S. saprophyticus *(two clinical isolates), *S. warnerii *(one clinical isolate, ATCC25614), *S. xylosus *(ATCC29971, ATCC35033), *S. schleiferi *(DSMZ4809), and *S. epidermidis *(two clinical isolates, ATCC14990, ATCC49134) were obtained for testing. Coagulase-positive staphylococcus *S. intermedius *(ATCC29663), *S. aureus *(four clinical isolates, ATCC29213), and MRSA were also included (three clinical isolates).

Clinical isolates and reference strains of *Staphylococcus *species were grown using the standard methodologies. Briefly, lyophilized bacterial strains were diluted by Luria-Bertani (LB) or tryptic soy broth. After dilution, nearly all bacterial species were grown on blood agar plates. The three exceptions were *S. epidermidis *ATCC14990 and *S. capitis *ATCC27840 that were both grown on tryptic soy agar plates, and *S. epidermidis *ATCC49134 that was grown on a nutrient agar plate. Culturing was performed under aerobic conditions with the exception of *S. saprophyticus*, which was grown under anaerobic conditions. All strains were incubated at 37°C for least 24 hours.

#### Blood cultures

Blood samples were drawn into aerobic and anaerobic blood culture bottles (BacT/Alert^®^, bioMérieux, France) and were incubated in the blood culturing equipment BacT/ALERT 3 D (bioMérieux) for up to 5 or 6 days, at which time they were reported as negative when no sign of micro-organism growth was detected. If during the cultivation period possible growth was observed by the blood culturing instrument, it was identified and reported according to CLSI guidelines http://www.clsi.org in the Department of Bacteriology, HUSLAB (Finland). The cultivation took 1–3 days, with a further 1–2 days culture needed for the identification of pathogen from a positive blood culture. In total, 186 blood cultures were collected between May 2007 and June 2007. These were used as references to evaluate the performance and feasibility of the assay with that of standard routine diagnostic testing. Of these, 146 were blood culture positive and 40 were blood culture negative.

#### Oxacillin resistance

The susceptibility to oxacillin of the staphylococcal species was determined by disc diffusion according to CLSI guidelines, using Mueller-Hinton II agar base (cat no 212257, Becton, Dickinson and Company, USA) and antibiotic discs (Oxoid, UK), incubated at +35°C. Minimal inhibitory concentrations (MIC) values for oxacillin were determined by E-tests (Biodisk, Sweden) on Mueller-Hinton agar supplemented with 4 percent NaCl, and incubated at +30°C.

### Dna Extraction

The extraction of DNA from clinical isolates and reference strains was carried out as follows: one bacterial colony was picked from the plate and suspended in 100 μl of PBS. After centrifugation (at 3000 rpm, for 3 minutes), the supernatant was discarded and the pellet was suspended in 100 μl of TE. Two heating steps of 95°C for five minutes were performed sequentially with a 2 minutes cooling step between them. Finally, the solution was centrifuged (at 13 000 rpm, for 10 minutes) and the supernatant containing DNA was collected. In the case of the blood culture samples, 100 μl of the samples were collected for DNA extraction. The DNA was extracted using an automated nucleic acid extraction instrument Nuclisens^®^easyMAG™ (bioMérieux, France) according to the manufacturer's protocol (Generic 1.0.6). The eluation volume was 55 μl. A negative control, *i.e*., sterile water was included in each test series.

### Dna Amplification and Labelling

The broad-range PCR primers gBF (5'-CGICCIGGKATGTAYATHGG-3') and gBR (5'-RMICCWACICCRTGYAGICCICC-3') were modified from primers introduced by Roth and colleagues (2004) [4]. We reduced the number of degenerated regions in primers by using inosines. The primers amplified a ~300 bp region of the bacterial *gyrB *and *parE *genes. In addition, specific primers for *mecA *gene, mecAR (5'-TTACTCATGCCATACATAAATGGATAGACG-3') and mecAF (5'-AATACAATCGCACATACATTAATA-3'), were designed. To enhance *S. aureus *amplification SaurF (5'-AGACCTGGTATGTATATTGG-3') and SaurR (5'-CCAACACCATGTAAACCACC-3') primers were further designed. All the reverse primers were biotinylated at their respective 5'-end.

The PCR reaction mixture contained 1 μM of gBF primer mixture (Metabion, Germany), 1 μM of biotin-labeled gBR primer mixture (Metabion, Germany), 0.165 μM of SaurF primer (Metabion, Germany), 0.165 μM of biotin-labeled SaurR primer (Metabion, Germany), 0.25 μM of mecAF primer (Metabion, Germany), 0.25 μM biotin-labeled mecAR primer (Metabion, Germany), 1× Hot Start Taq^® ^PCR buffer (Qiagen, Germany), in which the final concentration MgCl_2_was 2.0 mM, 300 μM of each of dNTP (Finnzymes, Finland), 1.5 g/l BSA (EuroClone, Italy), 0.125 U/μl Hot Start Taq^® ^DNA polymerase (Qiagen, Germany), 1.5 μl of isolated DNA, and water to bring the total volume to 15 μl. In the blood culture dataset, 1.5 μl of PCR control template was added in the reaction and the equivalent amount of water was reduced. A negative control, *i.e*., sterile water was included in each test series.

The PCR was performed using a Mastercycler^® ^ep*gradient S *thermal cycler (Eppendorf, Germany). The following PCR program was used: a denaturation step at 95°C for 15 minutes, 36 cycles of 10 seconds at 96°C, 35 seconds at 52°C, 10 seconds at 72°C, 5 cycles of 5 seconds at 96°C, 30 seconds at 65°C, 5 cycles of 5 seconds at 96°C and finally 30 seconds at 68°C. After the PCR, the success of the amplification of double-stranded DNA and single-stranded DNA was ascertained by gel electrophoresis using a 2% agarose gel containing SYBR^® ^Green II (Invitrogen, USA) or using Agilent BioAnalyzer (Agilent Technologies, USA).

### Microarray Construction

The microarray platform was an ArrayTube™ (Clondiag, Germany). The design of specific oligonucleotide probes were carried out according to the principles and methods described previously [4]. One to three different species-specific oligonucleotide probes were selected for each target species. In total, 22 species-specific probes for 12 bacteria, 2 CNS-specific, and 4 *mecA *resistance marker specific probes (Metabion, Germany) were chosen for spotting on the microarray (Table 1). All oligonucleotide probes were spotted as duplicates on the array. Two different oligonucleotides per spot were used for the *mecA *probes. Position control oligonucleotides containing a biotin label were attached to the array for verifying the correct function of the hybridization reagents.

### Hybridization and Scanning

The hybridization on microarray was performed as described previously [12] with only slight modifications. All incubation steps except that of the last precipitation reaction were performed under continuous agitation of 550 rpm at 25°C. Briefly, a first a prewash with 500 μl of water from 30 to 55°C for 5 to 10 minutes was done. Hybridization at 55°C for 10 minutes, of 1 μl of the biotinylated target and 99 μl hybridization buffer (250 mM Na_2_HPO_4_, 4.5% SDS, 1 mM EDTA, 1×SSC) took place on a microarray. When hybridization control oligonucleotides were included, they were added to the hybridization buffer. After hybridization, the microarray was washed in 500 μl of 0.2×SCC at 20°C for 5 minutes. Incubation with 100 μl of blocking buffer (2% milk powder, 6×SSPE, 0.005% Triton-X100) was performed for 5 minutes at 30°C. Then 100 μl of 1:5000 dilution of streptavidin-conjugated horseradish peroxidase in PBS was applied for 10 minutes at 30°C followed by a similar washing step as described above. Finally, 100 μl of 3, 3', 5, 5'-tetramethylbenzidine (TMB) analog (Seramun Grün; Seramun Diagnostica, Germany) was added for the precipitation reaction at 25°C for 10 minutes. Microarray images were generated by ATR-01 Reader (Clondiag).

### Data-Analysis

The array images were analyzed with the Prove-it™ Advisor software (Mobidiag, Finland, http://www.mobidiag.com). The software performed image analyses and result reporting, including the identification of the bacterial targets and the evaluation of the control probes. This took place automatically without user involvement in adjusting any of the parameters. The target identifications were made by software using multiple parameters such as signals from the probe oligonucleotides on the array. These were interpreted using built-in rules and parameters specific for each assay type. All the probes for a specific bacterial target were required to be positive for that target to be classified as positively identified, except for the CNS probes of which only 2 of 4 specific oligonucleotides were required to be positive. If both CNS and *S. epidermidis *probes in the analyses were positive, only *S. epidermidis *was reported due to its identification by species-specific probes. The original array layout contained spots, which were not included in the final probe panel. Microarray data files have been deposited in NCBI's Gene Expression Omnibus database and are accessible through GEO Series accession number GSE17221.

### Sequencing of CNS Samples

For sequencing of the CNS samples 16S_rRNA_F (5'-AGAGTTTGATCYTGGYTYAG-3') [25] and 16S_rRNA_R (5'CTTTACGCCCARTRAWTCCG-3') [26] were used as reported earlier. The primers amplified a ~550 bp region of the bacterial 16S rRNA genes. The PCR reaction mixture contained F and R primer mixture at a final concentration of 0.4 μM (Sigma, USA), 1× Hot Start Taq^® ^PCR buffer (Qiagen, Germany), in which the final concentration of MgCl_2 _was 2.0 mM, 200 μM of each of dNTP (Finnzymes, Finland), 0.8 g/l BSA (EuroClone, Italy), 0.05 U/μl Hot Start Taq^® ^DNA polymerase (Qiagen, Germany), 2.5 μl of isolated DNA, and water to bring total volume to 25 μl. The PCR was performed using a Mastercycler^® ^ep*gradient S *thermal cycler (Eppendorf, Germany). The PCR program was initialized by a 15 minute denaturation step at 95°C followed 36 cycles of 30 seconds at 95°C, 30 seconds at 54°C, and 30 seconds at 72°C. The PCR program ended with 10 minute step at 72°C. After the PCR, the success of the amplification of dsDNA was verified by gel electrophoresis using 2% agarose gel containing ethidiumbromide (Sigma, USA). The amplified PCR product was purified using the QIAquick^® ^PCR purification Kit (250) (Qiagen, Germany) and a minimum of 50 ng of product was mixed with either the forward or reverse primer (0.42 μM). Water was added to bring the total volume up to 12 μl. Sequencing was performed using cycle sequencing with Big Dye Terminator kit (version 3.1) supplied by Applied Biosystems (ABI, CA, USA) and the reactions were run on ABI 3130xl capillary sequencer according to the manufacturer's instructions.

Sequences were edited and analyzed with the Vector NTI Advance™ (Invitrogen, USA) and BioEdit http://www.mbio.ncsu.edu/BioEdit/bioedit.html programs using the ClustalW alignment algorithm version 1.4 [27]. We used the BLAST algorithm [28] to search for homologous sequences in the European Bioinformatics database and the National Center for Biotechnology Information database http://www.ebi.ac.uk/Tools; blast.ncbi.nlm.nih.gov/Blast.cgi).

### Statistical Analysis

We compared the results and calculated the sensitivity, specificity, and confidence interval (CI) values according to CLSI guidelines (EP12-A2, User protocol for evaluation of qualitative test performance, http://www.clsi.org. Briefly, these analyses were performed using the following definitions: true-positive (TP), true-negative (TN), false-negative (FN), and false-positive (FP). The sensitivity was calculated as follows: TP/(TP+FN), and the specificity was calculated as TN/(TN+FP).

## Authors' contributions

AKJ and MM drafted the manuscript. SL, PP, and AA performed the experiments in the laboratory. All authors participated in the concept development, read and approved the final manuscript.
